# Profiles of endogenous ABA, bioactive GAs, IAA and their metabolites in *Medicago truncatula* Gaertn. non-embryogenic and embryogenic tissues during induction phase in relation to somatic embryo formation

**DOI:** 10.1007/s00425-021-03582-8

**Published:** 2021-02-13

**Authors:** Ewa Kępczyńska, Anna Orłowska

**Affiliations:** Institute of Biology, University of Szczecin, Wąska 13, 71-415 Szczecin, Poland

**Keywords:** Abscisic acid, Bioactive gibberellins, Indole-3-acetic acid, Legume, Somatic embryogenesis

## Abstract

**Main conclusion:**

During the 3-week-long induction phase, when *M. truncatula* cells leaf explants from non-embryogenic genotype (M9) and embryogenic variant (M9-10a) were forming the callus, biosynthesis and degradation of ABA, Gas and IAA proceeded at different levels. Induction of embryo formation is related to a lower ABA content, compared to the content of IAA and that of total bioactive GAs.

**Abstract:**

Endogenous phytohormones are involved in the regulation of zygotic embryogenesis, but their role, especially of ABA, a plant growth inhibitor, in inducing somatic embryogenesis (SE) in angiosperms is still incompletely known. To arrive a better understanding of the ABA role in the process, we analyzed simultaneously and in detail changes in the contents of both ABA and five bioactive GAs (GA_4,_ GA_7,_ GA_1_, GA_3,_ GA_6_) and IAA in *M. truncatula* non-embryogenic M9 (NE) and embryogenic M9-10a (E) genotypes. The initial leaf explants of both genotypes, and particularly NE, contained many times more ABA compared to the total bioactive GAs or IAA. In tissues during the entire 21-day induction all the hormones mentioned and their metabolites or conjugates were present; however, their contents were found to differ between the lines tested. The ABA level in primary explants of NE genotype was more than two times higher than that in E genotype. An even larger difference in the ABA content was found on the last day (day 21) of the induction phase (IP); the ABA content in E callus was over six times lower than in NE callus. In contrast, the IAA and GAs contents in primary explants of both genotypes in relation to ABA were low, but the contents of IAA and GAs exceeded that of ABA in the M9-10a tissues on the last day of IP. It is shown for the first time that endogenous ABA together with endogenous bioactive GAs and IAA is involved in acquisition of embryogenic competence in *Medicago truncatula* leaf somatic cells. These findings have a strong functional implication as they allow to improve the SE induction protocol.

## Introduction

Somatic embryogenesis (SE), a nonsexual process, is the developmental restructuring of different somatic cell types along different embryogenic pathways that lead to regeneration of embryos capable of germinating to form complete plants (Zimmerman 1993; Pasternak et al. 2002; Fehér et al. 2003; Imin et al. 2004; Rose et al. 2013; Rose 2019; Fehér 2015). Its fundamental phases are characterized, at the morphological level, by the induction of proembryogenic structures followed by the somatic embryo formation and maturation leading to plant regeneration. Each of these phases is regulated by various physical and chemical factors the most critical of which is considered to be the exogenous application of plant growth regulators. Exogenous stimulants, such as auxins and cytokinins, are most commonly used in the form of their synthetic analogues such as the auxinic herbicide 2,4-dichlorophenoxyacetic acid (2,4-D) and kinetin. Knowledge on the role of endogenous classical plant growth phytohormones such as abscisic acid (ABA), a growth inhibitor, and growth stimulators: gibberellins (GAs) and indole-3-acetic acid (IAA) during induction of the somatic embryogenesis is far from satisfactory. These phytohormones play important roles during many phases of the plant life cycle and have a pleiotropic range of physiological effects. Their biosynthetic pathways start in plastids and end, just like their catabolism, in the cytoplasm.

Endogenous levels of ABA appear to be significant for the initiation of embryogenic cultures in some angiosperms. Kiyosue et al. (1992) and Jiménez and Bangerth (2001b), Guiderdoni et al. (1995), Jiménez and Bangerth (2000) and Nakagawa et al. (2001) found higher ABA levels in embryogenic callus (E) lines than in non-embryogenic (NE) ones in carrot, sugarcane, grapevine, and melon, respectively. However, the opposite was found in *Hevea brasiliensis* (Etienne et al. 1993) and in *Medicago falcata* (Ivanova et al. 1994), with E calli showing lower ABA levels than NE calli. In turn, Jiménez et al. (2005) suggested that ABA is not directly involved in the very initial stages of the embryogenic development of the carrot callus tissue, since the endogenous level of the hormone did not change in response to the presence/absence of 2,4-D. Thi and Pleschka (2005) reported a positive relationship between the endogenous contents of ABA in petioles and somatic embryogenesis of some *Daucus* species. In turn, no significant differences in the ABA content were observed between the embryogenic and non-embryogenic peach cotyledons used as initial explants (Pérez-Jiménez et al. 2013).

Kikuchi et al. (2006) reported the ABA carrot somatic embryo formation to be inhibited by the application of fluridone, an ABA biosynthesis inhibitor. The compound targets phytoene desaturase, involved in the carotenoid biosynthesis; some carotenoids are precursors of ABA biosynthesis (Gamble and Mullet 1986). Application of fluridone in our experiments on *Medicago sativa* SE, at the induction, proliferation or differentiation phase exerted a negative effect on the somatic embryo production and development by (Ruduś et al. 2009), which suggests that indirect somatic embryogenesis in alfalfa cultures requires some level of this plant growth inhibitor. Later, it was confirmed that the capacity of *Arabidopsis thaliana* embryogenic callus to form somatic embryos was strongly impaired by fluridone present in the somatic embryo-inducing medium (Su et al. 2013). Moreover, earlier, we have shown that fluridone decreased ABA levels in developing somatic embryos of *M. sativa* (Ruduś et al. 2009). Thus, the data obtained so far from experiments involving applications of both ABA and its biosynthesis inhibitor point to the participation of the endogenous ABA in SE regulation in angiosperm plants.

Most of the data related to the role of gibberellins in SE come mainly from studies on exogenous application of GA_3_ to different media at different stages of SE and point to between-species differences (Ruduś et al. 2002). Little information is still available on the contents of gibberellins during SE. Noma et al. (1982) detected several GAs (GA_1_, GA_4_, GA_7_) during carrot SE; these hormones were also found in undifferentiated cells from a non-embryogenic cell line. The very high levels of endogenous biologically active polar GAs in carrot cultures were associated with the absence of embryogenic development. On the other hand, significantly higher levels of endogenous GAs (GA_1_, GA_3_, GA_20_) were found in the embryogenic callus of maize, compared to those in the non-embryogenic callus (Jiménez and Bangerth 2001a). Furthermore, Jiménez and Bangerth (2000, 2001a, b) and Jiménez et al. (2005) found no difference between GAs levels in cultures of grapevine, carrot, and wheat showing different embryogenic characteristics. Previously, we showed that during a 3-week induction phase in *Medicago truncatula,* when the cells of the leaf explant from embryogenic (M9-10a) and non-embryogenic (M9) genotypes dedifferentiate to form callus, all the active gibberellins from 13-hydroxylation pathway (GA_1_, GA_3_, GA_6_) as well as from the non-13-hydroxylation pathway (GA_4_, GA_7_) are present (Igielski and Kępczyńska 2017). However, the contents of GA_3_ and also of GA_6_ were observed to increase in the M9-10a genotype as the induction phase progressed. Thus, the few studies referred to above, concerning endogenous GAs contents in cultures of non-embryogenic and embryogenic genotypes showed once again the ambiguity in the data.

The auxin IAA is considered to be the phytohormone most important for cell division and differentiation as well as for the induction of somatic embryogenesis (Pasternak et al. 2002). During direct SE in *M. falcata*, a fast embryogenic induction was correlated with high IAA and low ABA levels in the initial leaf explants (Ivanova et al. 1994). No significant differences were observed in IAA and, as mentioned above, in ABA contents between initial E and NE cotyledon explants of *Prunus persica* L (Pérez-Jiménez et al. 2013).

In turn, an ABA level higher than that of IAA was detected in the initial petiole explants used for SE induction in *M. sativa* cv. Rangelander (Ruduś et al. 2009). The level of endogenous IAA increased transiently in all the *M. sativa* subsp. *varia* protoplast cultures; the IAA accumulation was correlated with activation of the division cycle in embryogenic-type cells (Pasternak et al. 2002). The very low levels in callus and suspension, followed by quite constant but high levels in embryos developing from globular to the early cotyledonary stage was observed; afterwards, the IAA content decreased substantially in late-cotyledonary stage and declined further in mature embryos (Ruduś et al. 2009).

To date, there have been no data concerning the simultaneously occurring endogenous levels of ABA, biologically active GAs, IAA, and their catabolites or conjugates during the SE induction phase in plants, including *M. truncatula.* The competence to induce SE is known to be highly correlated with the genotype, as exemplified by two *M. truncatula* Gaertn. cv. Jemalong embryogenic variants, 2HA and M9-10a (Nolan et al. 1989; Neves et al. 1999; Araújo et al. 2004). These lines are considered to be a good model for the study of SE, because both are derived from non-embryogenic genotypes, A17 and M9, respectively. Therefore, it was considered justified to use embryogenic (M9-10a) and non-embryogenic (M9) lines of *M. truncatula* Gaertn. cv. Jemalong for comparing the endogenous levels of the hormones in tissues of these lines.

Drawing conclusions on the role of ABA, a classic growth inhibitor, in SE induction in plants is still hampered by the lack of exhaustive data. Nolan et al. (2014) stated that the synergism they observed between ABA and GAs as a result of their exogenous application to the medium at the beginning of the induction phase in *M. truncatula*, requires confirmation by analyzing their endogenous contents in explants.

Therefore, the present study was conducted to determine the levels of endogenous ABA and its catabolites in tissues of embryogenic (M9-10a) and non-embryogenic (M9) *M. truncatula* genotypes during the induction phase, and to compare it to the content of the classical growth stimulants such as bioactive GAs and IAA and their metabolites.

The results could provide new insight on the involvement of endogenous together with bioactive GAs and IAA in SE induction in plants, which can be used to improve protocols on the induction phase during which application of the phytohormones studied, at an appropriate concentration and time, can be considered. ABA is known at present to play important roles in the regulation of SE, mainly in the differentiation and maturation phases of somatic embryos.

## Materials and methods

### Plant materials

For mother plant production, we used seeds of two *Medicago truncatula* Gaertn.cv. Jemalong genotypes: the non-embryogenic genotype (M9) and the embryogenic variant (M9-10a), kindly provided by Professor Pedro Manuel Fevereiro, Instituto de Tecnologia Quimicae Biologica (ITQB), Portugal (Neves et al. 1999; Santos and Fevereiro 2002). Fresh seeds obtained from mother plants were ripened at 25 °C for 2 months and then stored in − 20 °C. Before sowing seeds were scarified using 96% H_2_SO_4_ for 8 min and then seeds were five times rinsed with cold sterile water. After scarification seeds were placed in sterile 15 cm Petri dishes (100 per one plate) on filter paper Whatmann moistened with water. The seeds were stratified in the dark at 4 °C for 2 days and then were transferred to 20 °C for 1 day to germinate. Seedlings with well-developed embryo radicle were placed in pots with a sterile mixture of sand, soil, perlite and vermiculite (1:1:1:1). Plants were grown in a growth room at 24/22 °C ± 1 °C day/night temperature, under a 16/8 h photoperiod of 120 µM m^−2^ s^−1^ Green LED (Philips) for 2 months. These mother plants were used for all experiments related to the role of hormones during SE induction in *Medicago truncatula*. Some of the obtained results concerning the role of gibberellins were published earlier (Igielski and Kępczyńska 2017), and the rest are described in this paper.

### Tissue culture protocol

The tissue culture involved the use of the non-embryogenic (M9) and embryogenic M9-10a genotypes of *M. truncatula* Gaertn. cv. Jemalong according to the protocol described previously (Igielski and Kępczyńska 2017). The initial explants (3 per dish) were prepared as squares of 1 cm × 1 cm size with one central cut perpendicular to the vascular bundles. For induction of SE and callus formation, leaf explants were cultured for 21 days in a Petri dish (ø 55 mM) on SH medium (Schenk and Hildebrandt 1972) supplemented with 0.5 µM 2,4-D, 1 µM zeatin and 3% (w/v) sucrose, pH 5.7 and solidified with 0.25% (w/v) gerlite. The cultures were maintained in climate chamber at dark and 28 °C. Subsequently, the 21-day-old callus tissues was transferred after weighting onto the MS medium (differentiation medium; Murashige and Skoog 1962) without hormones and kept in it for the next 21 days, following which the somatic embryos were counted.The callus development in leaf explants of both lines of *M. truncatula* after 2, 7, 14 and 21 days during the IP is in Fig. 2 of Igielski and Kępczyńska (2017).

### Endogenous hormone quantification

The endogenous ABA, GAs and IAA and their catabolites and conjugates were analyzed simultaneously in initial explants and tissues during the induction phase and callus development in the M9 and M9-10a genotypes on day 0, 2, 7, 14 and 21. Three biological replicates per time point were obtained. Each of the biological replicates consisted of 21 explants (three explants on each of the 7 Petri dishes). Samples were snap-frozen in liquid nitrogen and stored at − 80 °C until further analysis. The phytohormone contents were calculated as the amount per pmol/g fresh weight.

#### Analysis of ABA and its catabolites and conjugates

To quantify the endogenous level of ABA and its metabolites (phaseic acid, PA; dihydrophaseic acid, DPA; neophaseic acid, neoPA; 7′hydroxy-ABA, 7′OH-ABA; ABA-glucose ester, ABA-GE), the samples were prepared as above and processed after storage at − 80 °C. Contents of ABA and its metabolites were determined as described by Turečková et al. (2009). The plant tissues were homogenized and extracted for 1 h in 1 mL ice-cold methanol/water/acetic acid (10/89/1, by vol.). A mixture of internal standards containing 50 pmol of each ((−)-7′,7′,7′-^2^H_3_-phaseic acid; (−)-7′,7′,7′-^2^H_3_-dihydrophaseic acid; (−)-8′,8′,8′-^2^H_3_-neophaseic acid; (+)-4,5,8′,8′,8′-^2^H_5_-ABA-GE; (−)-5,8′,8′,8′-^2^H_4_-7′-OH-ABA and (+)-3′,5′,5′,7′,7′,7′-^2^H_6_-ABA) was added to the samples. The solutions were centrifuged (21,000*g*, 10 min, 4 °C) and the pellets were further re-extracted in the same way for 30 min. The combined extracts were purified by solid-phase extraction on Oasis^®^ HLB cartridges (60 mg, 3 mL, Waters, Milford, MA, USA), evaporated to dryness in a Speed-Vac (UniEquip), and finally analysed by UPLC-ESI(−/+)-MS/MS (Turečková et al. 2009).

#### Analysis of auxin and its catabolites and conjugates

Contents of auxin and its catabolites and conjugates in the plant samples were determined following the methods described by Pěnčík et al. (2009). Briefly, a 10 mg sample was extracted with 1 mL cold phosphate buffer (50 mM; pH 7.0) containing 0.02% sodium diethyldithiocarbamate with internal standards: [^13^C_6_]IAA, [^13^C_6_]oxIAA, [^15^N,^2^H_5_]IAAsp and [^15^N,^2^H_5_]indole-3-acetyl-glutamate (IAGlu). The samples were then centrifuged at 36,000*g* for 10 min, acidified with 1 M HCl to pH 2.7 and purified by solid-phase extraction (SPE) using C8 columns (Bond Elut, 500 mg, 3 mL; Varian). After evaporation under reduced pressure, the samples were analyzed for the auxin content using Acquity UHPLC™ (Waters) linked to a triple quadrupole mass detector (Xevo TQ MS™; Waters).

#### Determination of bioactive gibberellins and their major catabolites

To quantify the endogenous level of bioactive gibberellins (GA_4,_ GA_7,_ GA_1_, GA_3,_ GA_6_) and catabolites (GA_8,_ GA_34_), samples were prepared as above and analyzed for the GAs content according to Urbanová et al. (2013) with some modifications. A 20 mg tissue culture sample was homogenized in 2-mL Eppendorf tubes with 1 mL of 80% acetonitrile containing 5% formic acid after addition of the GA internal standards {[^2^H_2_]GA_1_, [^2^H_2_]GA_3_, [^2^H_2_]GA_4_, [^2^H_2_]GA_6_, [^2^H_2_]GA_7,_ [^2^H_2_]GA_8,_ [^2^H_2_] GA_34_ from OlChemIm, Olomouc, Czech Republic)} using an MM 301 vibration mill (http://www.retsch.de) at 27 Hz for 3 min, and 2-mM zirconium oxide beads were added to each tube to increase the extraction efficiency during homogenization. The tubes were then placed in a fridge (4 °C) and extracted overnight with stirring using a bench top laboratory rotator Stuart SB 3 (http://www.bibby-scientific.com). The homogenates were centrifuged at 36,000*g* for 10 min at 4 °C using Beckman Avanti™ 30 centrifuge (Beckman Coulter Inc., Brea, CA, USA). The supernatants were further purified using mixed-mode anion exchange cartridges (http://www.waters.com) and analysed by ultra-high performance liquid chromatograph (Acquity UPLC™ System; Waters) coupled to triple-stage quadrupole mass spectrometer (Xevo^®^ TQ MS, Waters MS Technologies, Manchester, UK) equipped with an electrospray interface (ESI). GAs were detected using multiple-reaction monitoring mode (MRM) based on the transition of the precursor ion [M–H]^−^ to the appropriate product ion. Data were acquired and processed by MassLynx™ 4.1 software (Waters, Manchester, UK) and GA levels were calculated using a standard isotope-dilution method (Rittenberg and Foster 1940).

### Statistical treatment

All the experiments were carried out in triplicate. Changes in embryo formation and contents of ABA, GAs and IAA and their metabolites were analysed using Statistica 13 (StatSoft, Kraków, Poland). The results are expressed as mean ± SD (*n* = 3). Statistical analyses were performed using the Student’s *t* test and one-way ANOVA followed by Tukey’s HSD post-hoc test. Differences between the mean values were considered to be significant at *P* < 0.01 or *P* < 0.05.

## Results

### Contents of ABA, bioactive GAs_,_ IAA and their main catabolites and conjugates in non-embryogenic M9 and embryonic M9-10a *M. truncatula* tissues during the induction phase

**Fig. 1:**
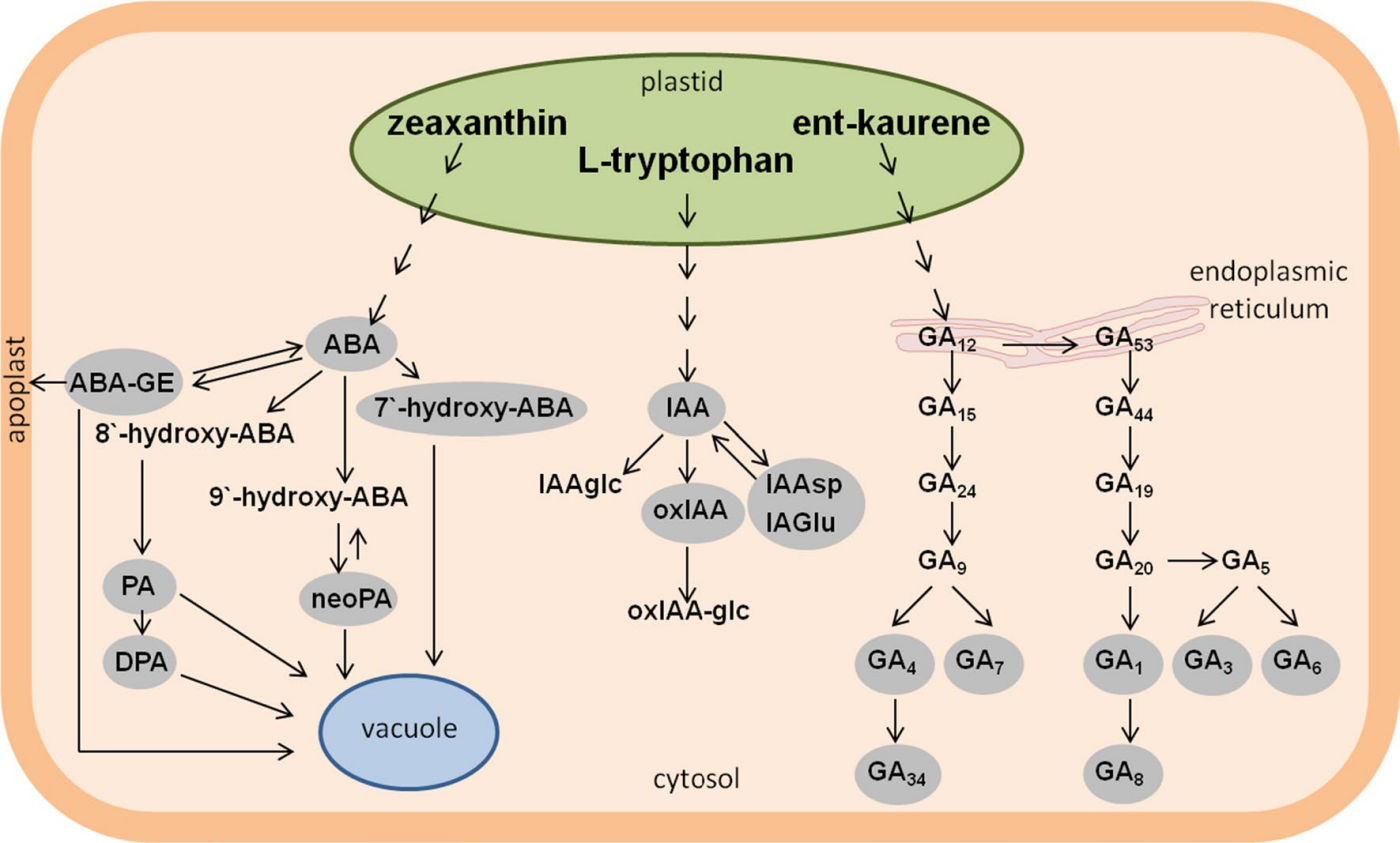
A schematic diagram illustrating the metabolic pathways of ABA, IAA and GAs analyzed in this study. The cellular location of metabolites in plastids, endoplasmic reticulum, cytoplasm and apoplast (the compounds analyzed in this work are shown as grey fields). *ABA* abscisic acid, *ABA-GE* abscisic acid glucosyl ester, *PA* phaseic acid, *DPA* dihydrophaseic acid, *neoPA* neo-phaseic acid, *IAA* indole-3-acetic acid, *IAAglc* glucosides of IAA, *oxIAA* oxindole-3-acetic acid, *oxIAA-glc* oxindole-3-acetyl-glucose, *IAAsp* indole-3-acetyl-aspartate, *IAGlu* indole-3-acetyl-glutamate, *GAs* gibberellins [Adapted from Hedden and Thomas (2006)]

Contents of endogenous ABA, GAs and IAA and their catabolites and conjugates were determined on day 0, 2, 7, 14 and 21 in initial leaf explants and in tissues. The metabolic pathways of the compounds analyzed are illustrated schematically in Fig. 1.

### ABA levels and its catabolites

**Fig. 2:**
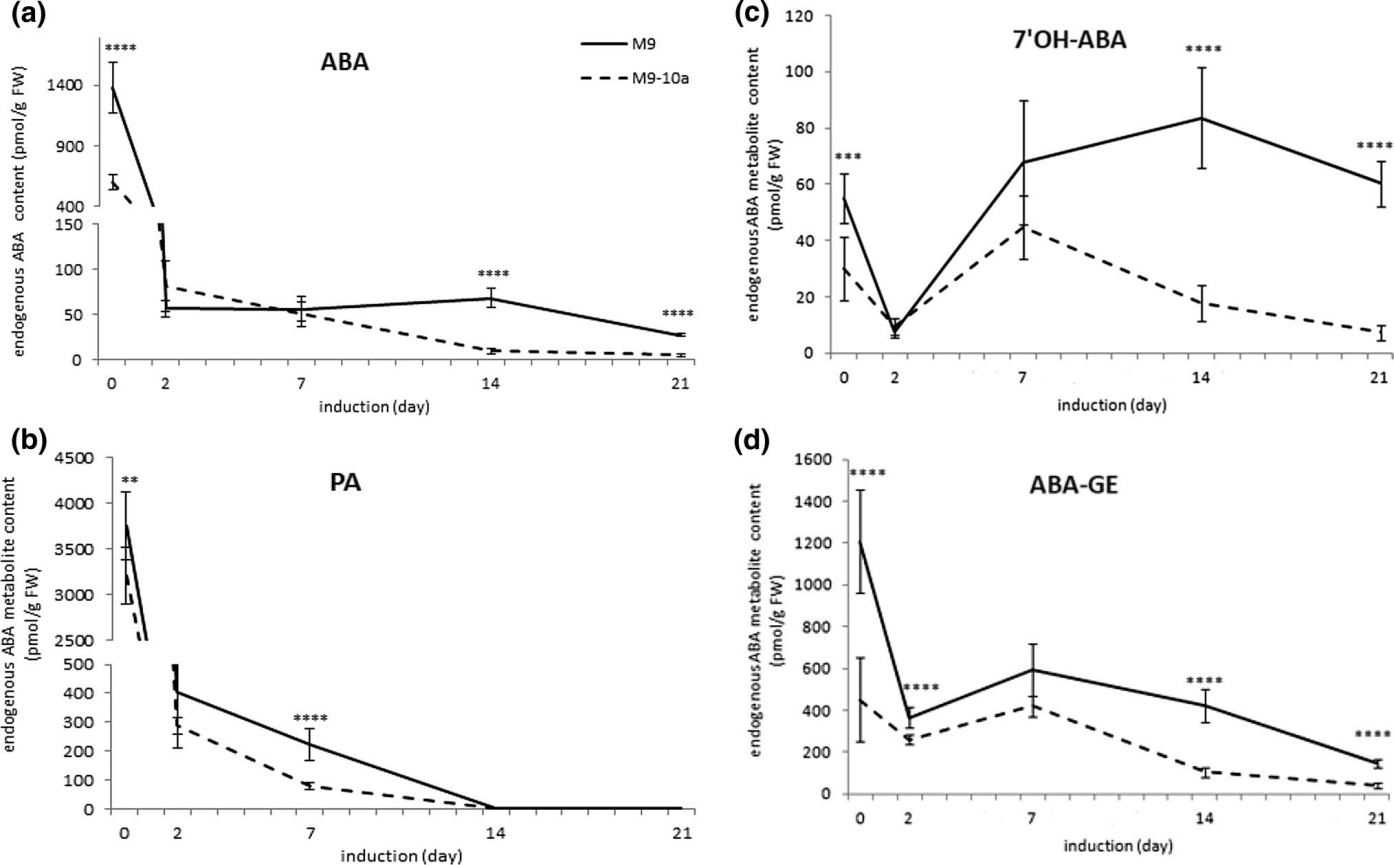
Contents of ABA (**a**), its conjugate (**b**) and catabolites (**c**, **d**) in *M. truncatula* non-embryogenic (M9) and embryogenic (M9-10a) genotype tissues on day 0, 2, 7, 14 and 21. Statistical analyses: two-tailed *t* test with 0.05 confidence interval. Asterisks represent significance levels: *P ≤ 0.05, **P ≤ 0.01, ***P ≤ 0.001 and ****P ≤ 0.0001. Bars indicate ± SD (*n* = 3). DPA—ND. neoPA—ND

As the capacity for SE initiation and somatic embryo production from leaf explants in the M9-10a genotype was phase during which leaf cells are dedifferentiated and the callus tissue develops. The endogenous contents of ABA and its catabolites ABA glucosyl ester (ABA-GE), phaseic acid (PA), dihydrophaseic acid (DPA), neophaseic acid (neoPA) and 7′hydroxy-ABA (7′OH-ABA) were assayed in initial explants and in tissues of both the embryogenic M9-10a and non-embryogenic M9 genotypes of *M. truncatula* (Figs. 1, 2). In primary explants (day 0) of both lines, of all the compounds analyzed, PA, the major ABA catabolism product, occurred at the highest amounts; the PA contents in M9 and M9-10a explants were about 3750 and 3198 pmol/g FW, respectively (Fig. 2b). The ABA level in primary explants of NE genotype was 2.3 times higher than that in the E genotype; in the NE and E lines, the contents were about 1381 and 595 pmol/g FW, respectively (Fig. 2a).

7′OH-ABA and ABA-GE were also present in explants on day 0, their levels being 1.8 and 2.7 times higher, respectively, in the NE genotype compared to the E genotype (Fig. 2c, d). On day 2, the levels of ABA, PA and 7′OH-ABA were similar in explants of both genotypes, and only the level of ABA-GE in the NE explants proved significantly higher (by a factor of 1.5) than that in the E genotype. On day 7, the explants of both M9 and M9-10a genotypes, on which callus had already appeared (Fig. 2 of Igielski and Kępczyńska 2017), showed their ABA levels (55.42 and 49.93 pmol/g FW, respectively) not to differ significantly. Similarly, there were no significant differences in the ABA-GE and 7′OH-ABA levels between tissues of two lines, the differences being significant in the PA level as the NE explants showed the PA contents three times higher than those in the E line. A week later (day 14), PA was not detected in tissues of both lines, while the content of ABA, ABA-GE and 7′OH-ABA in the M9 genotype were significantly higher (by a factor of almost 8, 4 and 5, respectively) than those in the M9-10a genotype. On the last day of the induction phase (day 21), when the callus tissues of both genotypes were transferred to the differentiation medium, the tissues were found to contain ABA, ABA-GE and 7′OH-ABA. The ABA content in the E tissues was more than six times lower than that in NE (26.18 and 4.12 pmol/g FW, respectively). The M9 callus showed contents of ABA-GE and 7′OH-ABA to be 3.7 and 8.3 times higher, respectively, than those in M9-10a. Of all the ABA catabolites tested, shown in the diagram (Fig. 1), neither neoPA nor DPA was detected in the primary explants or in the tissues of both genotypes during the entire induction process.

### Bioactive GAs and their main catabolites

**Fig. 3:**
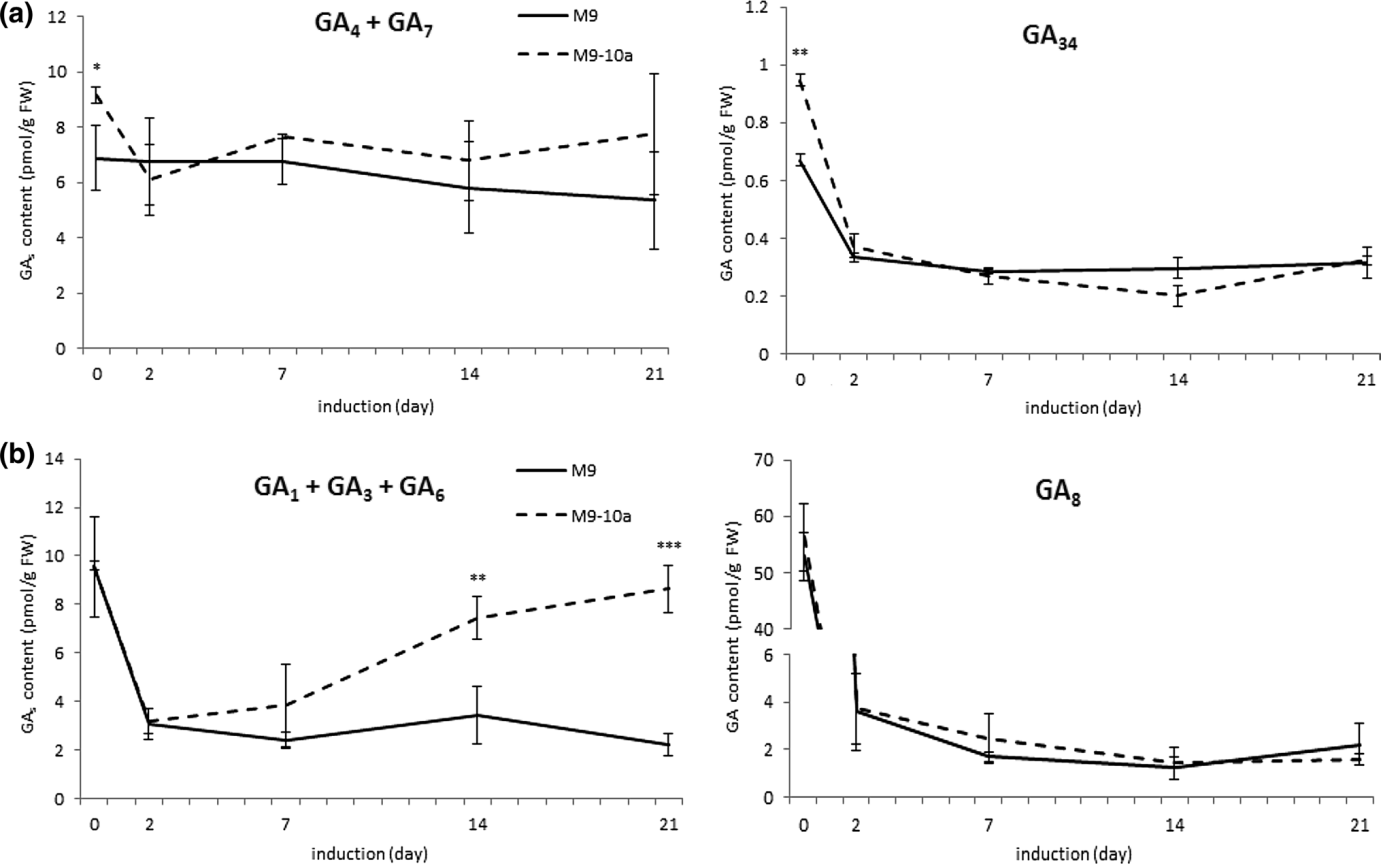
Contents of GA_4_ + GA_7_ and GA_34_ from non-13-hydroxylation (**a**), GA_1_ + GA_3_ + GA_6_ and GA_8_ from 13-hydroxylation (**b**) pathways in *M. truncatula* non-embryogenic (M9) and embryogenic (M9-10a) genotype tissues on day 0, 2, 7, 14 and 21. Statistical analyses: two-tailed *t* test with 0.05 confidence interval. Asterisks represent significance levels: *P ≤ 0.05, **P ≤ 0.01, ***P ≤ 0.001 and ****P ≤ 0.0001. Bars indicate ± SD (*n* = 3)

Our earlier research (Igielski and Kępczyńska 2017) showed that during a 3-week long induction phase, when the cells of the leaf explants from M9 and M9-10a genotypes were forming calli, all the bioactive GAs from the non-13-hydroxylation (GA_4_, GA_7_) and 13-hydroxylation (GA_1_, GA_3_, GA_6_) pathways were present, but it was only for a few of them that differences in the contents between the genotypes were found. The study mentioned above demonstrated that among the bioactive gibberellins detected, it was mainly the content of GA_3_ and also—to a lesser extent—the level of GA_6_, both originating from GA_5_, that increased and appeared to be connected with the acquisition of embryogenic competence. However, it should be emphasized that the tissues of both genotypes at all the time points tested contained all the bioactive gibberellins and their metabolites. Therefore, in the present paper, which continues to present results obtained from an extensive experiment dealing with the analysis of endogenous phytohormone levels during IP and in embryogenic and NE tissues, we are showing (Fig. 3) the total contents (sums) of gibberellins from their both metabolic pathways (Fig. 3). There were no significant differences in the total content of GA_4_ + GA_7_ from the non-13-hydroxylation pathway in the tissues of the NE and E genotypes at all the IP time points, except for the initial explants; primary explants of the M9-10a line showed somewhat higher contents (Fig. 3a). GA_34_ (a GA_4_ catabolite) was found in both M9 and M9-10a; its content was higher in primary explants of the E genotype then in NE, but on days 2, 7 and 21 of the IP, the tissue levels in both variants were identical. In turn, the total contents of the polar bioactive GAs of the 13-hydroxylation pathway (GA_1_ + GA_3_ + GA_6_) in the initial explants of both genotypes were similar (about 9.5 pmol/g FW) (Figs. 1, 3b). On day 2 and 7, there were no significant differences in the total content of the three GAs in the tissues of both the M9 and M9-10a genotypes. However, on days 14 and 21, the contents did differ; the M9-10a line callus showed the contents to be more than two and almost four times higher, respectively, than the contents in M9.

GA_8_ (a GA_1_catabolite) was detected in both lines as well (Figs. 1, 3b). Its levels in the primary M9 and M9-10a explants were very high (above 53 pmol/g FW). After 2, 7, 14 and 21 days, there were no significant differences in the content between the M9 and M9-10a tissues. In 21-day-old calli, the levels were 2.2 and 1.62 pmol/FW, respectively.

### Levels of IAA and its catabolites and conjugates

**Fig. 4:**
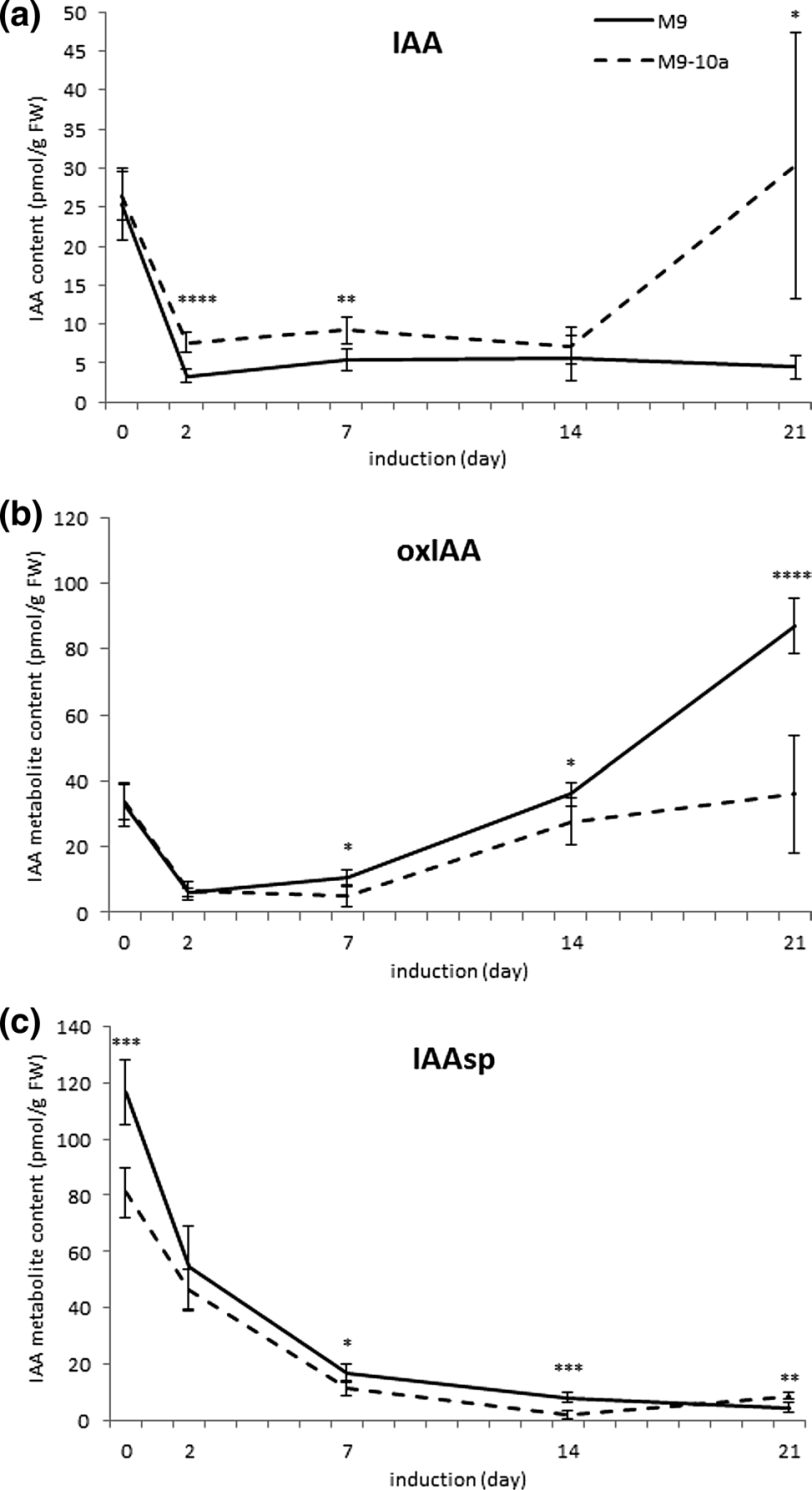
Contents of IAA (**a**) and its catabolite (**b**) and conjugate (**c**) in *M. truncatula* non-embryogenic (M9) and embryogenic (M9-10a) genotype tissues on day 0, 2, 7, 14 and 21. Statistical analyses: two-tailed *t* test with 0.05 confidence interval. Asterisks represent significance levels: *P ≤ 0.05, **P ≤ 0.01, ***P ≤ 0.001 and ****P ≤ 0.0001. Bars indicate ± SD (*n* = 3). IAAglu—ND

Since auxins are known to be crucial for SE induction and progress, it was justified to trace their synthesis and catabolism during SE induction by comparing their contents during the callus formation in the NE and E genotypes (Fig. 4a). The primary NE and E explants showed similar IAA levels, just above 25 pmol/g FW. On day 2 and 7 of IP, the IAA content in the E tissues was 2.3 and 1.7 times higher, respectively, compared to the contents in the NE genotype. On day 21, the last day of IP, the IAA content in the M9-10a callus was about six times higher than that in the M9 callus.

In the primary and 2-day-old explants of both genotypes, the contents of oxIAA, an IAA catabolite, did not differ (Fig. 4b). However, the contents of the catabolite in the NE line on days 7, 14 and 21 were higher by a factor of 2.2, 1.3, and almost 2.4, respectively than those in the E line.

IAAsp, an IAA conjugate, was detected in tissues of both line at all the time points tested (Fig. 4c). Its amount in the primary explants of the NE genotype was higher (116.59 pmol/g FW) than that in the E line’s primary explants (80.93 pmol/g FW). On day 21, when the calli were transferred to the differentiation medium, the NE and E genotypes contained 4.59 and 8.73 pmol/g FW, respectively.

Indole-3-acetyl-glutamate (IAGlu), another amino-acid conjugate, was not detected in the primary explants and tissues during the entire 21-day IP.

### Ratio of ABA level to the levels of total bioactive GAs and IAA

**Fig. 5:**
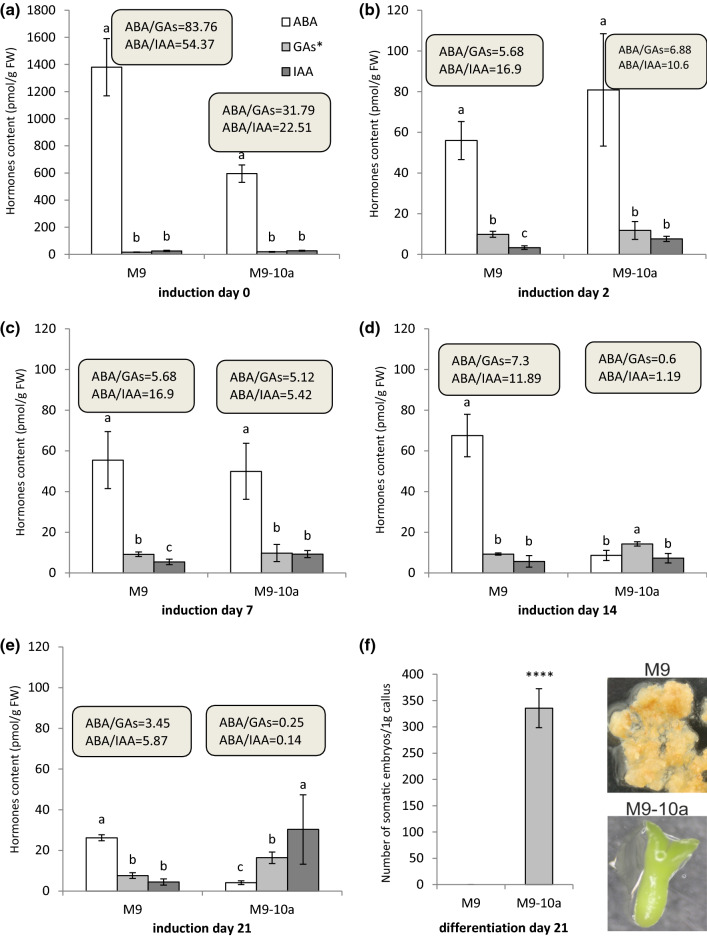
**a**–**e** Comparison of contents of ABA, total bioactive GAs* (GA_4_ + GA_7_ + GA_1_ + GA_3_ + GA_6_) and IAA in *M. truncatula* non-embryogenic (M9) and embryogenic (M9-10a) genotype tissues on day 0, 2, 7, 14 and 21. Grey frame, comparison of ratios between contents of ABA and those of total bioactive GAs* and between ABA and IAA. **f** Ability to produce somatic embryos in calli of M9 and M9-10a genotypes. **a**–**e** Statistical analyses were performed using one-way ANOVA with 0.05 confidence interval and Tukey’s HSD post-hoc test. Bars indicate ± SD (*n* = 3). **f** Statistical analyses: two-tailed *t* test with 0.05 confidence interval. Asterisks represent significance levels at ****P ≤ 0.0001. Bars indicate ± SD (*n* = 3)

To arrive at a final conclusion regarding the role of ABA, a plant growth inhibitor, and IAA and GAs growth stimulants, in the SE induction, their contents in the E and NE line tissues were compared. Thus, their contents in the initial explants and in tissues of both genotypes were simultaneously compared at five time points over the course of IP (Fig. 5). In addition, the ABA to IAA and ABA to GAs ratios were calculated because, as mentioned earlier, all the bioactive gibberellins from both biosynthetic pathways (GA_4_, GA_7_, GA_1_, GA_3_, GA_6_) were present in the tissues of both lines, for which reason their contents were pooled. The initial explants contained all the phytohormones studied, but ABA accounted for most of their pooled content in both lines (1380.84 and 595.52 pmol/g FW in the NE and E line, respectively) compared to the contents of GAs (16.47 and 18.80 pmol/g FW, respectively) and IAA (25.38 and 26.45 pmol/g FW, respectively). The differences in the contents of the hormones are very well illustrated by the inhibitor/stimulant ratios (Fig. 5a; ratios are in darkened fields). After two days, the ABA content clearly decreased, by a factor or almost 25 and 7 in the NE and E genotypes, respectively, and was still significantly higher than the GAs and IAA contents (Fig. 5b). The IAA content in the E line tissues was twice that in the NE. A week later, the ratios of the hormones in the NE genotype did not change but decreased in the E line (Fig. 5c). After 14 days, the ABA, GA, and IAA contents showed clear differences (Fig. 5d). In the NE line callus the ABA content was still higher than the contents of both stimulants, but the ratios were lower. On the other hand, the ABA level in the E line callus equalled that of IAA, while the GA content was higher than that of ABA. On the last day of IP (day 21), the NE callus ABA content exceeded the GA and IAA contents (Fig. 5e). Meanwhile, the E line callus showed the proportions of the hormones tested to change radically in favor of growth stimulants. The ABA content was as low as 4.12 pmol/g FW, whereas the contents of GAs and IAA were higher by a factor of 4.3 and 7.3, respectively. Thus, the transfer of 21-day-old M9 and M9-10a calli from the SH induction medium to the MS differentiation medium resulted in the embryo formation in the E line callus only after 21 days in the medium (Fig. 5f).

It is demonstrated for the first time that of key importance for the SE induction in *M. truncatula*a is a higher content of plant growth stimulants, such as auxin and bioactive GAs, than that of ABA, a classical plant growth inhibitor.

## Discussion

Although the past two decades witnessed much progress in the understanding of the function of exogenous (auxin and cytokinin) and endogenous (ethylene, ABA, jasmonates and GAs) hormones during somatic embryogenesis in angiosperm plants (reviewed by Rose et al. 2010, 2013; Méndez -Hernández et al. 2019; Rose 2019), knowledge on the role of ABA, a classical plant growth inhibitor, in relation to IAA and GAs, classical plant growth stimulants in the initiation of the process remains very incomplete.

### The metabolism of ABA, bioactive GAs and IAA differs between the E and NE tissues of *M. truncatula* during the SE induction phase

#### ABA metabolism

Since we showed earlier that endogenous ABA is involved in the regulation of embryogenic callus growth and embryo production in embryogenic *Medicago sativa* cv. Rangelander (Ruduś et al. 2009), it was crucial to check its levels and its catabolites in embryogenic tissues of the M9-10a genotype during the 21-day-long IP and to compare them with tissue levels in the non-embryogenic M9 genotype. The ABA content in the initial explants of the NE genotype was more than twice that in the E genotype. However, the calli obtained from these explants, when transferred to the differential medium (day 21), contained over six times more of this growth inhibitor than the E calli, so the remarkably higher callus mass observed in M9-10a compared to M9 (Igielski and Kępczyńska 2017) was probably associated with lower ABA levels in that callus (Fig. 2). On the other hand, Kiyosue et al. (1992) who used ELISA for the ABA assay, reported a different pattern in carrot SE. After 14 days of induction, they detected over 67-fold more ABA in the embryogenic cells than in the non-embryogenic ones. Also, Jiménez and Bangerth (2001b), using radio-immunoassay with polyclonal antibodies, found tenfold more ABA in the E compared to the NE carrot callus. In turn, Pérez-Jiménez et al. (2013) showed no significant differences in ABA content between the embryogenic and non-embryogenic *Prunus persica* L. Batsch cotyledons used as initial explants. Perhaps these differences in the ABA contents between the tissues of embryogenic and non-embryogenic *M. truncatula* genotypes during the IP are due to the different content of ABA catabolites and conjugates. There are several metabolic pathways by which ABA can be removed or degraded in plant tissues as a means of a further regulation of ABA concentrations (Nambara and Marion-Poll 2005; Finkelstein 2013). ABA can be metabolized by oxidation, reduction or conjugation (Fig. 1). Contents of ABA derivatives (ABA-GE, PA, 7′OH-ABA) detected in *M. truncatula* initial explants were higher in the M9 than in the M9-10 genotype. Of the three catabolites (ABA-GE, PA, 7′OH-ABA), the PA content decreased the fastest, because no PA was found in the tissues of both genotypes after 14 and 21 days. These results seem to suggest that in both lines of *M. truncatula* the 8′-hydroxylation pathway is the main oxidation pathway of ABA breakdown. PA is sometimes further reduced to form dihydrophaseic acid (DPA), as was shown during the seed development of legumes such as chickpea and lentil (Slater et al. 2013). During the IP, DPA was not detected in tissues of the *M. truncatula* NE and E genotypes. ABA is regarded as a bioactive form, but PA has a faint ABA-like activity (Kepka et al. 2011). Also, 7′ and 9′-hydroxy ABA showed biological activity in *Brassica napus* embryos (Jadhav et al. 2008). The tissues of both lines showed the presence of 7′OH-ABA, however, the NE genotype tissues, both in the initial explants and after 7, 14 and 21 days, contained more 7′OH-ABA than the E tissues. Among several conjugated catabolites, the most widespread is glucosyl ester (ABA-GE) (Finkelstein 2013). Although it has been regarded as an inactive pool of ABA accumulated in the vacuole or apoplast (Fig. 1), ABA-GE is considered as storage or long-distance transport form (Jiang and Hartung 2008). Among all the ABA catabolites tested and detected in tissues of both the NE and E genotypes of *M. truncatula* during the induction phase, the ABA-GE content was the highest, particularly in the NE tissues. ABA production from ABA-GE, mediated by *β*-glucosidase present in vacuoles is also considered a key pathway for regulating the local ABA concentration in response to environmental stress (Xu et al. 2012). The results presented in this work clearly demonstrate that during the 21-day-long IP in M9 and M9-10a tissues of *M. truncatula* the ABA the metabolism changes dynamically. To answer the question whether the changes in the ABA content are accompanied by changes in contents of growth stimulants, GAs, IAA and their metabolites, their contents were determined in the same tissues.

#### Bioactive GAs metabolism

In contrast to that of ABA, a possible role of GAs in inducing SE has received little attention, although biologically active GAs are known to be present during SE (Jiménez and Bangerth 2001a; Jiménez et al. 2005; Igielski and Kępczyńska 2017). There are contrasting opinions on the GAs involvement in SE, as evidenced by data produced by experiments in which exogenous GAs, and their biosynthetic inhibitors, including paclobutrazol, were used. Paclobutrazol, a triazole inhibitor of GAs biosynthesis, when present in the induction medium, strongly inhibited the callus growth and subsequent production of somatic embryos in *M. truncatula* embryogenic M9-10a, which suggested that endogenous GAs are required for both these processes, and the reduction of their levels results in SE impairment (Igielski and Kępczyńska 2017). Previously, we observed that paclobutrazol present in the *M. sativa* induction medium also reduced both the callus growth and embryo formation (Ruduś et al. 2002). By analyzing the expression of all genes participating in biosynthesis and catabolism of GAs and metabolite contents in tissues of the NE and E genotypes of *M. truncatula* during the whole IP, we demonstrated that all bioactive GAs are present in tissues of both genotypes and that mainly GA_3_ of all the bioactive GAs detected appeared to be associated with the acquisition of embryogenic competence (Igielski and Kępczyńska 2017). In the present study, there was a need to show detailed data on the total content of all bioactive GAs arising in two pathways of their biosynthesis to answer the question of their level at individual stages of the IP. This will allow a comparison with the ABA level. With these analyses, we are able to meet the expectations of Prof. Rose’s group, as articulated in 2014 (Nolan et al. 2014). It has been suggested that a low exogenous ABA:GA ratio enhanced SE in *M. truncatula* embryogenic line 2HA, but it is necessary to analyze the actual intracellular bioactive GAs and ABA levels. Therefore, we performed such analyses for the first time. In our earlier paper, we showed that during a 21-day-long IP, when cells of leaf explants from embryogenic (M9-10a) and non-embryogenic (M9) genotypes of *M. truncatula* were forming the callus, all the bioactive GAs from non-13-hydroxylation (GA_4_ and GA_7_) and 13-hydroxylation (GA_1_, GA_3_ and GA_6_) pathways were present, but the contents of only a few of them differed between the genotypes tested (Igielski and Kępczyńska 2017). The results presented here clearly show no significant differences in the total level of two GAs from the non-13-hydroxylation pathway (GA_4_ + GA_7_) in the tissues of both M9 and M9-10a on IP day 0, 2, 7, 14 and 21, but there were differences between these genotypes when the total level of the three GAs (GA_1_ + GA_3_ + GA_6_) synthesized via the second pathway was compared (Fig. 3). With time of induction, the E tissues showed specific activation of the 13-hydroxylation pathway resulting from an increase in the total level of the three GAs mentioned. Jiménez and Bangerth (2001a) reported that the contents of GAs (GA_1_ + GA_3_ + GA_20_) in the 7-week-old embryogenic callus of maize were significantly higher than in the non-embryogenic line, which partially agrees with our results. There were no significant differences in the content of the main catabolites, GA_34_ and GA_8_, in tissues of both lines after 2, 7, 14 and 21 days of IP.

#### IAA metabolism

Since the IAA is the main phytohormone for the SE induction in many plants, its content and products of its conjugation and degradation during the SE induction in *M. truncatula* were also analyzed. Although no differences were found in the IAA content in the initial explants of both genotypes, the content of this growth stimulator in tissues of the E genotype after 2, 7, and especially after 21 days of IP was higher than that in the NE genotype. Differences in the IAA contents between the E and NE tissues may result from different degradation of this hormone. The plant cell IAA level is known to depend not only on the de-novo synthesis, but it can be reduced by conjugation (mainly with amino acids and sugars) or degradation (Rosquete et al. 2012). The presence of the amino acid conjugate IAAsp and the major IAA catabolite oxIAA was detected in tissues of both M9 and M9-10a; IAAglu, another important IAA conjugate, was not present. It is worth noting that the primary explants of both genotypes contained definitely more IAAsp and oxIAA than IAA. In turn, in 21-day-old NE callus the highest level of oxIAA was found. Some IAA conjugates can be regarded as storage forms that can be hydrolyzed to form free IAA (Ludwig-Müller 2011), but the oxidative attenuation of IAA through formation of the catabolite, oxIAA, is considered the major pathway for IAA inactivation (Zhao et al. 2013). Ayil-Gutiérrez and coworkers (2013) suggested that a balance among free IAA and its amide conjugates is necessary to allow the expression of the embryogenic potential during the induction of *Coffea canephora.*

### Functional implication of changing the ratios of ABA to GA and IAA

To the best of our knowledge, this is the first study in which endogenous contents of ABA, total bioactive GAs and IAA and their main catabolites and conjugates were simultaneously measured in embryogenic and non-embryogenic cells during the IP of SE in angiosperm plants. All the results presented indicate that biosynthesis and degradation of the phytohormones analyzed occur in the tissues of both M9 and M9-10a genotypes of *M. truncatula* during the 21-day IP. Tissues of both M9 and M9-10a at individual stages of development differ in their contents of these hormones, most importantly, the ratio of the ABA content to the total content of total five bioactive GAs (GA_4_, GA_7_, GA_1_, GA_3_, GA_6_) and to IAA changes, as illustrated in Fig. 5. The ABA level in the tissues of the NE genotype at all the time points was higher than the contents of GAs and IAA, which is clearly illustrated by the ratios of ABA to GAs and ABA to IAA. The 21-day-old calli of the embryogenic genotype M9-10a contained more GAs and IAA in relation to ABA. When these calli were transferred to the differentiation medium, embryos were formed in the M9-10a calli, no embryo formation taking place in the M9 calli.

In conclusion, the results presented may suggest that ABA participates in the induction of embryogenesis in *M. truncatula*. A higher ABA content in primary explants of non-embryogenic genotype (M9) compared to the content in primary explants of the embryogenic variant (M9-10a) may indicate that high ABA levels reduce embryogenic competence in *M. truncatula*. The ABA content lower than that of active GAs and IAA at the time of callus transfer to a hormone-free differentiation medium enables the formation of embryos.

These results show that two genotypes of the same species (*M. truncatula*) and the same variety (Jemalong) differ in their ABA, GAs and IAA metabolism. These findings also have a strong functional implication as they allow to improve the SE induction protocol in *Medicago truncatula*, an important crop model for legumes, the third largest family of higher plants and second to cereals in the area harvested and total production (Gepts et al. 2005). The study of legume embryogenesis is important for human nutrition. It is worth noting here that embryogenesis has been extensively studied in *Arabidopsis*, but the plant is not a legume and the details of embryogenesis are not identical to those of *Medicago*. Knowledge about changing hormone levels about of ABA, GAs, IAA and theirs catabolites, over the course of the IP of *M. truncatula* SE, can be further applied to investigate the crosstalk between the signaling pathways that influence these phytohormones. Earlier we showed the inhibitory effect of manipulation of GAs metabolism during the IP, by applying exogenous GA_3,_ which not only impaired the production of somatic embryos but also significantly decreased expression of *Medicago truncatula BABY BOOM*, SE marker gene (Igielski and Kępczyńska 2017). In different species the expression of *PICKLE*, a negative regulator of SE is probably related to differences in how the hormone networks optimize its expression. In *Arabidopsi*s, down-regulation of this gene expression is linked to high exogenous ABA:GA ratio (Henderson et al. 2004), and low ABA:GA ratio in *M. truncatula* (Nolan et al. 2014).

#### *Author contribution statement*

 EK initiated and designed research interpreted the results and wrote the manuscript. AO participate in tissue samples collection, performed statistical analysis of the results and their graphic development.

## Abbreviations

7′OH-ABA: 7′Hydroxy-ABA
E: Embryogenic genotype
ABA-GE: Abscisic acid glucosyl ester
DPA: Dihydrophaseic acid
IAAsp: Indole-3-acetyl-aspartate
IP: Induction phase
NE: Non-embryogenic genotype
oxIAA: Oxindole-3-acetic acid
PA: Phaseic acid
SE: Somatic embryogenesis
